# Point-of-care devices: the next frontier in personalized chemotherapy

**DOI:** 10.4155/fsoa-2017-0059

**Published:** 2017-07-14

**Authors:** Maria Amélia Carlos Souto Maior Borba, Carlos Henrique Madeiros Castelletti, José Luiz de Lima Filho, Danyelly Bruneska Gondim Martins

**Affiliations:** 1 Molecular Prospection & Bioinformatics Group (ProspecMol) - Laboratory of Immunopathology Keizo Asami (LIKA), Federal University of Pernambuco (UFPE), Av. Prof. Moraes Rego 1235, 50670–901, Cidade Universitária, Recife, PE, Brazil; 2 Agronomic Institute of Pernambuco (IPA), Av. General San Martin 1371, 50761–000, Bongi, Recife, PE, Brazil; 3 Biochemistry Department, Federal University of Pernambuco (UFPE), Av. Prof. Moraes Rego 1235, 50670–901, Cidade Universitária, Recife, PE, Brazil

**Keywords:** cancer, chemotherapy, point-of-care devices, precision medicine

The ‘standard-of-care’ is the commonest type of treatment, determined by averaging responses across large cohorts. However, every patient has their own genetic background and lifestyle, which indicates that each one should receive individualized care as based on clinical trials. The ‘Precise Medicine’ era has emerged to achieve successful treatment for each patient based on their personal characteristics and it is pushing forward ‘point-of-care’ (POC) devices [1]. The POC concept relies on portable, user-friendly and robust devices to perform sensitive and specific detection anywhere. This technology can also be linked to a cell phone coupled to a detection device [2]. One of the prime fields for application of this technology is cancer therapy, focusing on evaluating the treatment scheme for each patient.

## Point-of-care devices

The global population has grown and the maximum age has been raised together with cancer incidence. The world-wide landscape of cancer treatment costs US$ 2 trillion/year [3], which impacts not only public programs but also the patient budget [4]. Besides, chemotherapy does not always achieve the expected results [5,6], so the patient relapses and the treatment costs increase. Combined systems of drug delivery and biosensing are breaking paradigms in the timeline from the clinical investigation of symptoms to starting the treatment. As an example, patch nanotechnology has shown promising results with doxorubicin delivery and also in diabetes and chronic viral infections fields [7]. Therefore, the concept of using POC devices goes beyond the clinician's office; it achieves patient empowerment.

An additional advantage in POC testing is the possibility of monitoring disease progression through specific biomarker-based dosage and monitoring treatment effectiveness, in real time, by dosing drug and metabolites ratios. Real-time patient monitoring can be integrated with wireless data collection and analysis, as observed for a POC device dedicated to acute stress that sends the vital signs to mobile applications (US FDA approved) [8]. The possibilities for similar devices in the clinical oncology routine are endless and they are also near to becoming a reality.

Computational techniques have been used for prospecting drug targets in metabolic pathways based on the humongous amount of human genome data available. New tools have emerged to reduce the computational costs, creating accessible and intuitive resources and allowing the use of systems biology insight. The computational approach is the best to perceive the individual phenotype and then develop a therapeutic approach. Other *in silico* tools evaluate how a point mutation affects the protein's function and its intermolecular interactions on three dimensional simulation software, which allows the understanding of protein–drug interaction, an important application in pharmaceuticals industries [9,10]. These predictive computational methodologies are a prebench step that saves time and reduces the costs of the *in vitro* tests, being crucial to cancer research due to the disease's complexity.

## Chemoresistance & precision medicine

Each tumor has its own characteristics, composed of its particular and heterogeneous pool of cells. Once exposed to chemotherapy, drugs kill the sensitive cells but a small set of cells may be resistant to therapy and allows a clonal expansion that changes the tumor characteristics and determines treatment failure. On the other hand, genetic and epigenetic alterations lead to extrinsic resistance that occurs secondarily to exposure to the drug [11]. Both intrinsic and extrinsic resistance are traceable through POC devices using specific biomarkers. However, to develop precise and efficient POC devices, it is mandatory to understand how cancer cells evade therapy.

Among the mechanisms of cellular resistance to treatment, some are universal and others are disease related. A classic universal drug resistance mechanism is increased drug efflux, mediated by the overexpressed proteins of the ATP-binding cassette family, also known as multidrug resistance and P-glycoprotein [12]. An example of disease-related resistance is observed in luminal breast cancer treated with tamoxifen, a selective modulator of the estrogen receptor. The tamoxifen nonresponsive patients may have: dysfunction in tamoxifen metabolism, such as *CYP2D6* gene polymorphisms, the major metabolizing enzyme on CYP450; or polymorphisms on the estrogen receptor or its downstream effectors [13]. Another example in breast cancer was observed in a study that evaluated 14 metastatic sites of a breast cancer patient harboring *PIK3CA*-activating mutations. In those foci that presented BYL719-resistance, *PTEN* mutations were identified, evidencing a selective therapeutic pressure that conferred to the tumor temporal heterogeneity [14].

The so-called ‘n of 1’ approach arises to overcome these mechanisms that lead to chemotherapy failure, exploring a new interpretation of clinical trial results [15]. This comprises looking for the exceptional responders – the individuals that were different to the majority and achieve optimal responses in failed clinical trials. On the other hand, it is the nonresponders that can also contribute to revealing new possible approaches because, potentially, their genomes hold the keys to solving the therapeutic paradigm of promising *in vitro* studies that fail in clinical trials. These studies highlight the need to review the drug testing methods to create a precision-driven approach. Rather than consider the patient as a number in a thousand, it is necessary to observe and consider genetics and environmental factors [16]. Upon the application of this reasoning, the use of POC devices will allow monitoring patient response and drug metabolism efficiency, thus leading to more accurate conclusions at the trials.

Not that long ago cancer diagnosis was a death sentence. Nowadays this reality has changed, but the survival expectancy upon cancer diagnosis is still often given in months and is potentially influenced by the treatment choices. Going forward, in the technological era, it is not acceptable that clinicians have to test the best therapy instead of initiating the treatment under accurate molecular evaluation. So, what is keeping us from the next step? We need to recruit professionals, such as bioinformaticians and computer scientists, clinicians, biomedical engineers and pharmaceuticals to work in this field to empower the patient, so they will not be a simple number at statistics, but a whole and complex individual.
